# Effect of *Helicobacter pylori*-Associated Chronic Gastritis on Autonomous Activity and Sleep Quality in Mice

**DOI:** 10.3389/fphar.2022.785105

**Published:** 2022-02-04

**Authors:** Haihua Liu, Wenlong Zheng, Ling Zhang, Tangtang Lin, Yang Tang, Ling Hu

**Affiliations:** ^1^ Institute of Gastroenterology, Science and Technology Innovation Center, Guangzhou University of Chinese Medicine, Guangzhou, China; ^2^ First Affiliated Hospital of Gannan Medical University, Gannan Medical University, Ganzhou, China; ^3^ Shangyou Hospital of Traditional Chinese Medicine, Ganzhou, China

**Keywords:** autonomous activity, chronic gastritis, *Helicobacter pylori*, mouse behavior, sleep quality

## Abstract

Many reports have shown that patients with Hp-associated chronic gastritis exhibit anxiety and poor sleep quality. However, less is known about the effects and specific manifestations of Hp-associated chronic gastritis on autonomous activity and sleep quality in animals. Here, we investigated the effect of *Helicobacter pylori* (Hp)-associated chronic gastritis on autonomous activity and sleep quality in mice. To do this, a Hp-associated chronic gastritis mouse model was first established, then analyzed for autonomous activity, relative to controls, for 15 min using an autonomous activity tester. Next, sleep quality of mice was detected by sodium pentobarbital-induced sleep experiment and results compared between groups. The results showed that male mice in the model group exhibited higher activity counts but lower forelimb lift counts, relative to those in the control group, although there were no significant differences (all *p* > .05). Conversely, female mice in the model group recorded lower activity counts, albeit at no significant difference (*p* > .05), and significantly lower counts of forelimb lift (*p* < .05), relative to those in the control group. Notably, male mice in the model group had longer sleep latency and shorter sleep duration than those in the control group, albeit at no significant differences (all *p* > .05). On the other hand, female mice in the model group recorded significantly longer sleep latency as well as shorter sleep duration compared to those in the control group (all *p* < .01). We conclude that Hp-associated chronic gastritis exerts certain effects on autonomous activity and sleep quality of mice in a gender-dependent manner. Notably, female mice with Hp-associated chronic gastritis had lower activity and forelimb lift counts, as well as prolonged sleep latency, and shortened sleep duration. These effects were all statistically significant except for activity counts.

## Introduction

Chronic gastritis is a chronic inflammatory disease of the gastric mucosa characterized by various etiologies (Spużak et al., 2020), one of which is *Helicobacter pylori* (Hp) (Nagy et al., 2016; Kalach et al., 2021). Previous studies have shown that prevalence, grade and severity of chronic gastritis increases with the degree of Hp infection (Tiwari et al., 2020; Chitapanarux et al., 2021). Many clinical studies have shown that patients with Hp-associated chronic gastritis exhibit anxiety and poor sleep quality (Buzás, 2006; Takeoka et al., 2017; Kim et al., 2020), but the specific characteristics and pathological mechanisms remain unclear. Therefore, relevant experimental research should be carried out. However, the effects and specific manifestations of Hp-associated chronic gastritis on autonomous activity and sleep quality in animals remain unclear, due to scarcity of specific experimental studies. In the present study, we adopted a previously established Hp-associated chronic gastritis mouse model (Lee et al., 1997) to explore the effect and specific manifestations of Hp-associated chronic gastritis on autonomous activity and sleep quality in mice. Furthermore, we verified whether the observed effects were consistent with that in the human body, with the aim of laying a foundation for future experimental studies.

## Materials and Methods

### Experimental Animals

A total of 100 specific pathogen-free (SPF) C57BL/6 mice (male and female, 5–6 weeks old, 18–22 g) were purchased from Beijing Vital River Laboratory Animal Technology Co., Ltd., license number: SCXK (Beijing) 2016–0006. Animal certificate number: No.110011201110226373, No.11011201110226426. Mouse fodder was purchased from Shanghai Puluteng Biotechnology Co., Ltd. (lot number: P1101F-25-20201103020). The experimental protocol used herein was approved by the Ethics Committee of First Affiliated Hospital of Gannan Medical University.

### Hp SS1 Strain Culture

Hp culture medium was prepared with Columbia blood agar 23.4 g, brain-heart leaching powder 9.6 g and deionized water 780 ml, and autoclaved 20 mim at 121°C. Firstly, freeze 1 tube of Hp SS1 strain glycerin was taken from ultra-low temperature refrigerator (−80°C) and resuscitated on four Hp culture plates in the tri-gas incubator at 37°C for 48 h. Then, the Hp resuscitated in the previous step was transferred to new Hp culture plates and placed in the tri-gas incubator at 37°C for 48 h. Finally, an appropriate amount of 0.85% NaCl solution was used to elute the Hp growing on the plates (about 20–30 plates were needed), and the Hp solution was diluted to 3 × 10^9^ cfu/ml (about 30 ml). The concentration of Hp solution was quantified by hemacytometric counting and the Hp solution was prepared and used on the same day.

### Model Construction and Specimen Collection

Four mice, two males and two females, were randomly selected from 100 mice after quarantine, and subjected to 24 h of fasting prior to detection. The mice were sacrificed via cervical dislocation, under a biosafety cabinet, then sterilized surgical instruments used to remove their stomach tissues. These tissues were cut along the great bend of the stomach and longitudinally divided it into two parts. One part was placed in urease reagent, and the other was frozen with liquid nitrogen to await DNA extraction and polymerase chain reaction (PCR) detection. The remaining 96 mice were randomly divided into a model group (*n* = 24) and control group (*n* = 72), according to weight and gender. Firstly, mice in both groups were intragastrically administered with 0.1 mol/L NaHCO_3_ 0.5 ml/mouse in the second-level biosafety cabinet. After 1 h, mice in the model group were intragastrically injected with 3 × 10^9^ cfu/ml Hp SS1 bacterial solution in 0.85% NaCl solution 0.5 ml/mouse, while those in the control group were intragastrically injected with an equal volume of 0.85% NaCl solution. Food and water were forbidden for 12 h, prior to intragastric administration, and allowed 4 h after treatment. The above operations were performed once every 2 days for a total of five times. Twelve weeks following end of intragastric administration, 72 mice were selected from the control group and randomly divided into 8 groups according to their gender and weight. Among them, 6 groups (8 mice/group) were used for the grope experiment, comprising suprathreshold and subthreshold doses of sodium pentobarbital-induced sleep, whereas the remaining two groups (12 mice/group) were used for analysis of autonomous activity by the autonomous activity tester (Chengdu Techman Technology Co., LTD., model: ZZ-6) and detection of sleep quality by sodium pentobarbital-induced sleep experiment. On the other hand, 24 mice in the model group were randomly divided into two groups (12 mice/group) according to gender and weight, then two groups subjected to analysis of autonomous activity by the autonomous activity tester and detection of sleep quality by sodium pentobarbital-induced sleep experiment. After detection, 48 mice were sacrificed *via* cervical dislocation in a biosafety cabinet, their gastric tissues removed, and cut along the greater curvature of the stomach into two portions. One portion subjected to detection using the Hp Detection Kit (Urease), while the other was immediately fixed in 10% neutral formalin buffer, embedded in paraffin, sectioned, subjected to HE staining and observed by the digital slice scanner (TissueGnostics, model: TissueFAXS Plus).

### Indicator Detection

#### Evaluation of Hp-Associated Chronic Gastritis

Hp infection in gastric tissues was detected using PCR and urease test. PCR was performed targeting the 16S ribosomal RNA, using the following primer sequences that amplify a 375 bp fragment to detect murine *Helicobacter pylori* prevalent in experimental mouse colonies: 16S-Forward: TATGACGGGTATCCGGC, and 16S-Reverse: ATT​CCA​CTT​ACC​TCT​CCC​A. For the urease test, gastric tissue samples were incubated in an oven at 37°C. A color change, to red, indicated positive Hp infection. Gastric tissues from four mice randomly selected after quarantine were tested for Hp *via* PCR and urease test to exclude Hp infection in mice used in the experiment. After 12 weeks of modeling, mice in the model group were subjected to urease test to detect Hp colonization. Next, the gastric tissues were dehydrated and embedded in paraffin, sectioned and stained with HE, followed by the digital slice scanner to observe pathological changes associated with chronic gastritis according to the Sydney system (Dixon et al., 1996). The rate of Hp colonization and pathological changes of chronic gastritis were combined to evaluate the model of Hp-associated chronic gastritis.

#### Analysis of Autonomous Activity

A total of 24 mice in the control, and 24 in the model groups were selected and subjected to analysis of autonomous activity. Summarily, the mice were placed in an autonomous activity tester, during daytime, in a manner that both groups alternated. The mice were allowed to adapt to the test conditions, for 5 min, then the number of autonomous activity and forelimb lift counted over a period of 15 min. The autonomous activity tester was thoroughly cleaned, after each experiment, to remove stool, urine and other wastes, so as not to affect the next experiment.

#### Sleep Quality Detection

##### Direct Sleep Experiment

A total of 24 mice in the control and 24 in the model groups were selected for the direct sleep experiment. The righting reflex was considered to be normal if the mouse immediately returned to normal posture when placed in flat dorsal supine position. If the mouse failed to righting in 1 min, the righting reflex disappeared and the mouse went to sleep. Recovery of righting reflex indicated that the test animal was awake. In addition, the time taken between disappearance of the righting reflex and recovery was denoted sleep duration. The number of falling asleep and sleep duration of mice were recorded.

##### Grope Experiment of Suprathreshold and Subthreshold Doses Using Sodium Pentobarbital-Induced Sleep

We selected six control groups for evaluation of suprathreshold and subthreshold doses of sodium pentobarbital-induced sleep in mice. One day before the experiment, the mice were subjected to 16 h of fasting, but were allowed to drink water. The exploratory dose of sodium pentobarbital were of varying concentrations, namely 55, 50, 45, 40, 35 and 30 mg/kg, while the dose and volume of the first intraperitoneal injection were 40 mg/kg and 10 ml/kg, respectively. The dose of sodium pentobarbital was adjusted to determine the next dose according to the general situation of mice as well as the number of mice falling asleep within 30 min of administration. If none of the eight mice fell asleep, the sodium pentobarbital dose was the formal subthreshold dose of sodium pentobarbital-induced sleep. If all eight mice slept, the dose of sodium pentobarbital was the formal supramental dose of sodium pentobarbital-induced sleep.

##### Analysis of Subthreshold Dose of Sodium Pentobarbital-Induced Sleep

One control and one model group, comprising 12 mice each were selected for analysis of subthreshold dose of sodium pentobarbital-induced sleep. The mice were subjected to 16 h of fasting, a day prior to the experiment, but allowed drinking water. The mice were intraperitoneally injected with sodium pentobarbital (10 ml/kg) according to the determined subthreshold hypnotic dose. Failure of mice to righting reflex in 1 min was considered the criterion of falling asleep. The number of mice falling asleep within 30 min in each group was recorded and compared.

##### Analysis of Suprathreshold Dose of Sodium Pentobarbital-Induced Sleep

Similarly, 1 control (12 mice) and 1 model group (12 mice) were selected for analysis of suprathreshold dose of sodium pentobarbital-induced sleep. The mice were subjected to fasting, as previously described, then intraperitoneally injected with sodium pentobarbital (10 ml/kg). The mice were placed in a supine position, then immediately observed and tested for changes in righting reflex. Failure of righting reflex in 1 min was regarded as the criterion of falling asleep, whereas the period from the peritoneal injection of sodium pentobarbital to disappearance of righting reflex was considered the time of sleep latency. The period between the disappearance of righting reflex and recovery was sleep duration. The number of sleepless mice was recorded after 30 and 60 min, if any of the mice did not fall asleep, followed by calculation of sleepless rate. Sleep latency and sleep duration induced by sodium pentobarbital were observed and compared between groups.

### Statistical Analysis

All data were entered into EXCEL 2003 for statistical analysis, and presented as means ± standard deviation (SD). A Chi-square test was used to compare count data between suprathreshold and subthreshold doses of pentobarbital sodium-induced sleep in mice and the formal experiment of the subthreshold dose of sodium pentobarbital-induced sleep in mice. Data obtained from the autonomous activity observation and analysis of suprathreshold dose of sodium pentobarbital-induced sleep in mice were analyzed by using inter-group analysis of variance (F-test), then compared between groups. A Student *T*-test (unpaired) was used when the variance between groups was uniform. Otherwise, the corrected Student *T*-test was applied when variance between groups was not uniform. Statistical significance was set at *p* < .05.

## Results

### Model Establishment

**FIGURE 1:**
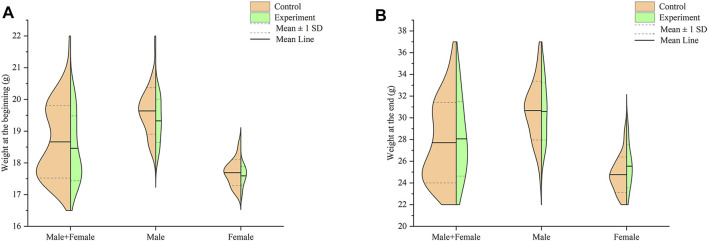
Effect of Hp-associated chronic gastritis on weight in mice. **(A)** Split violin plot showing the effect of Hp-associated chronic gastritis on weight at the beginning; **(B)** Split violin plot showing the effect of Hp-associated chronic gastritis on weight at the end.

Compared with the control group, the fur of the experiment mice became drier and less shiny overall. In terms of the weight of all mice at the end of the study, as shown in Figure 1, there was no significant difference between the experiment group and the control group at the beginning and end (*p* > .05).

**FIGURE 2:**
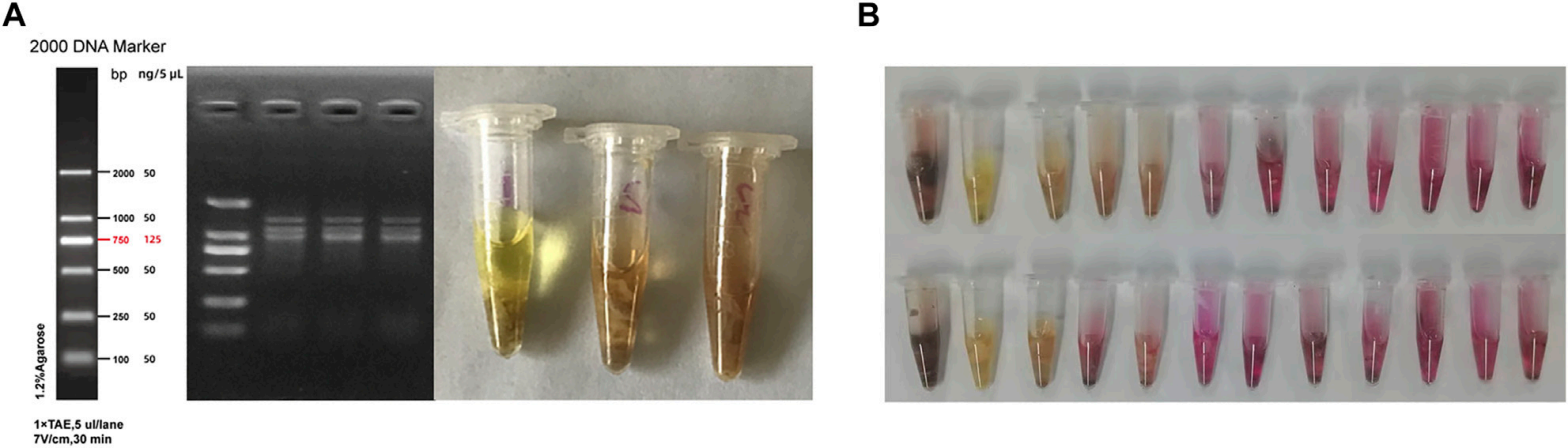
Hp colonization in the stomach tissues of mice. **(A)** Results from PCR and urease test after quarantine; **(B)** Results of urease test in the model group after 12 weeks of modeling.

Among four mice that were randomly selected for gastric tissue collection after quarantine, 1 was eliminated due to improper anatomical operation, while the remaining three were negative for Hp following PCR and urease test (Figure 2A). Moreover, results from the urease test showed that 70.8% of the mice in the experiment group were Hp positive after 12 weeks of modeling (Figure 2B).

**FIGURE 3:**
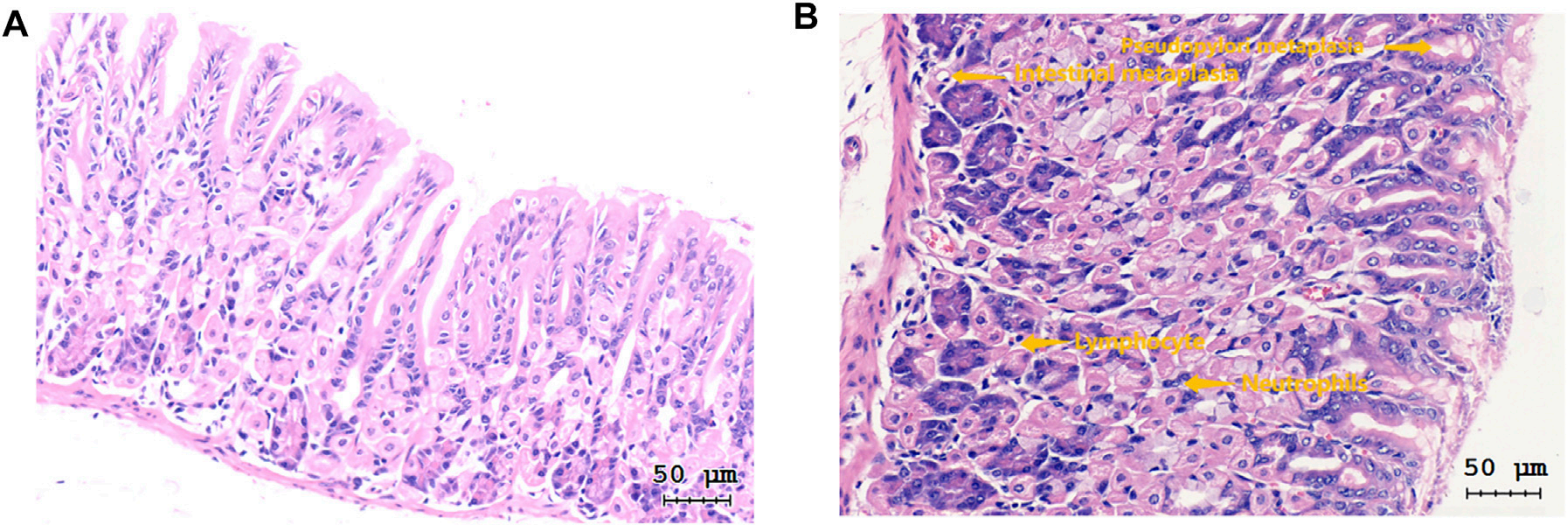
Images of HE staining of gastric tissue sections (magnification = ×200). **(A)** Representative image of HE staining of gastric tissue sections in the control group; **(B)** Representative image of HE staining of gastric tissue sections in the experiment group.

**FIGURE 4:**
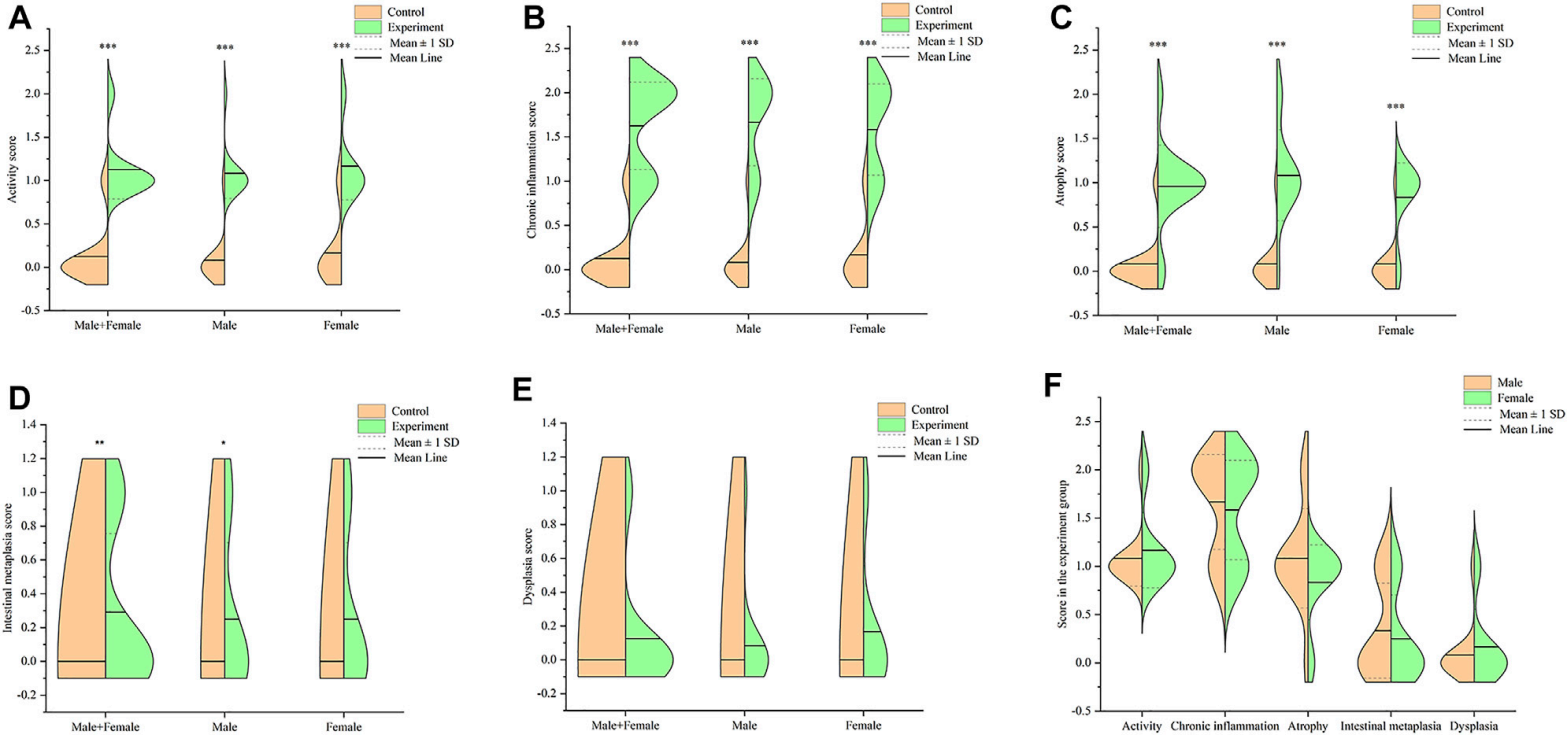
Pathological score in the stomach tissues of mice. **(A)** Split violin plot showing the activity score; **(B)** Split violin plot showing the chronic inflammation score; **(C)** Split violin plot showing the atrophy score; **(D)** Split violin plot showing the intestinal metaplasia score; **(E)** Split violin plot showing the dysplasia score; **(F)** Split violin plot showing the gender difference of pathological score in the experiment group. *p < .05, **p < .01, ***p < .001 versus control.

Notably, mice in the experiment group exhibited several pathological changes compared to those in the control group. These changes included infiltration of lymphocyte and neutrophils in the gastric mucosa layer, erosion, local necrosis of gastric mucosa epithelium, capillary hyperemia, edema and exudation, decreased gastric mucosa folds, as well as decreased and atrophic inherent glands of gastric mucosa (Figure 3). Some gastric tissues had mild intestinal metaplasia and a few had mild dysplasia. All mice used in the formal experiment of autonomous activity and sleep were taken for pathological score, as shown in Figure 4. There were significant differences in activity, chronic inflammation and atrophy scores between the experimental group and the control group. Due to the small quantity and mild degree of intestinal metaplasia and dysplasia, there was no significant difference, except for male mice in intestinal metaplasia. In the experimental group, there was no significant difference in the above pathological scores between male and female mice. These pathological changes indicated successful establishment of a Hp-associated chronic gastritis mouse model.

### Autonomous Activity

**FIGURE 5:**
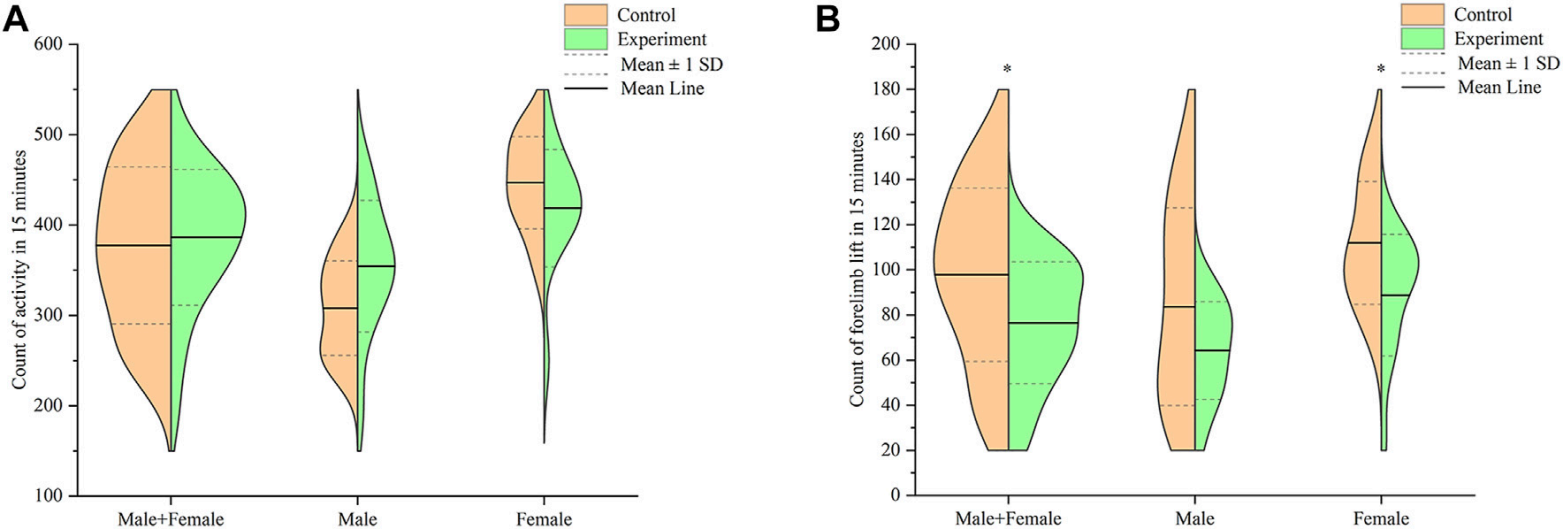
Effect of Hp-associated chronic gastritis on autonomous activity in mice. **(A)** Split violin plot showing the effect of Hp-associated chronic gastritis on the count of activity; **(B)** Split violin plot showing the effect of Hp-associated chronic gastritis on the count of forelimb lift. *p < .05 versus control.

Results of the comparison in activity counts between mice in the control and model groups, within 15 min, is shown in Figure 5A. Summarily, mice in the model group exhibited higher activity counts than those in the control group, although the increase was not statistically significant (*p* > .05). Male mice in the model group had higher activity counts than those in the control group, was albeit at no statistical significance (*p* > .05). Conversely, female mice in the model group exhibited lower activity count compared to those in the control group, although this was not significantly different (*p* > .05).

Results of the comparison in forelimb lift counts between mice in the control and model groups, within 15 min, are shown in Figure 5B. Summarily, mice in the model group recorded significantly lower forelimb lift counts than those in the control group (*p* < .05). Male mice in the model group had lower forelimb lift counts compared to those in the control group, albeit at no statistical significance (*p* > .05). On the other hand, female mice in the model group recorded significantly lower forelimb lift counts than their counterparts in the control group (*p* < .05).

### Sleep Quality

#### Direct Sleep Experiment

The count of falling asleep and sleep duration of mice in control group and model group were 0.

#### Optimal Suprathreshold and Subthreshold Doses of Sodium Pentobarbital-Induced Sleep

Results of the count of falling asleep and sleep rate after intraperitoneal injection of sodium pentobarbital over 30-min observation period are shown in Tables 1, 2. From the results, it was evident that the optimal suprathreshold and subthreshold doses of sodium pentobarbital-induced sleep were 55 and 35 mg/kg, respectively.

**TABLE 1:** Grope of suprathreshold dose of sodium pentobarbital-induced sleep.

Group	Count of mice	Count of falling asleep	Sleep rate (%)
55 mg/kg	8	8	100
50 mg/kg	8	7	87.5
45 mg/kg	8	7	87.5

**TABLE 2:** Grope of subthreshold dose of sodium pentobarbital-induced sleep.

Group	Count of mice	Count of falling asleep	Sleep rate (%)
45 mg/kg	8	7	87.5
40 mg/kg	8	4	50
35 mg/kg	8	0	0

#### Optimal Subthreshold Dose of Sodium Pentobarbital-Induced Sleep

Results of sleep induction in mice intraperitoneally injected with 35 mg/kg sodium pentobarbital over a 30-min observation period are shown in Table 3. One mouse in the control group fell asleep, while none was observed to sleep in the model group. Notably, we found no significant differences between the two groups with regards to sleep induction (*p* > .05).

**TABLE 3:** Effect of Hp-associated chronic gastritis on the experiment of subthreshold dose of sodium pentobarbital-induced sleep.

Group	Count of mice	Count of falling asleep	Count of not falling asleep
Control	12	1	11
Experiment	12	0	12

#### Optimal Suprathreshold Dose of Sodium Pentobarbital-Induced Sleep

**FIGURE 6:**
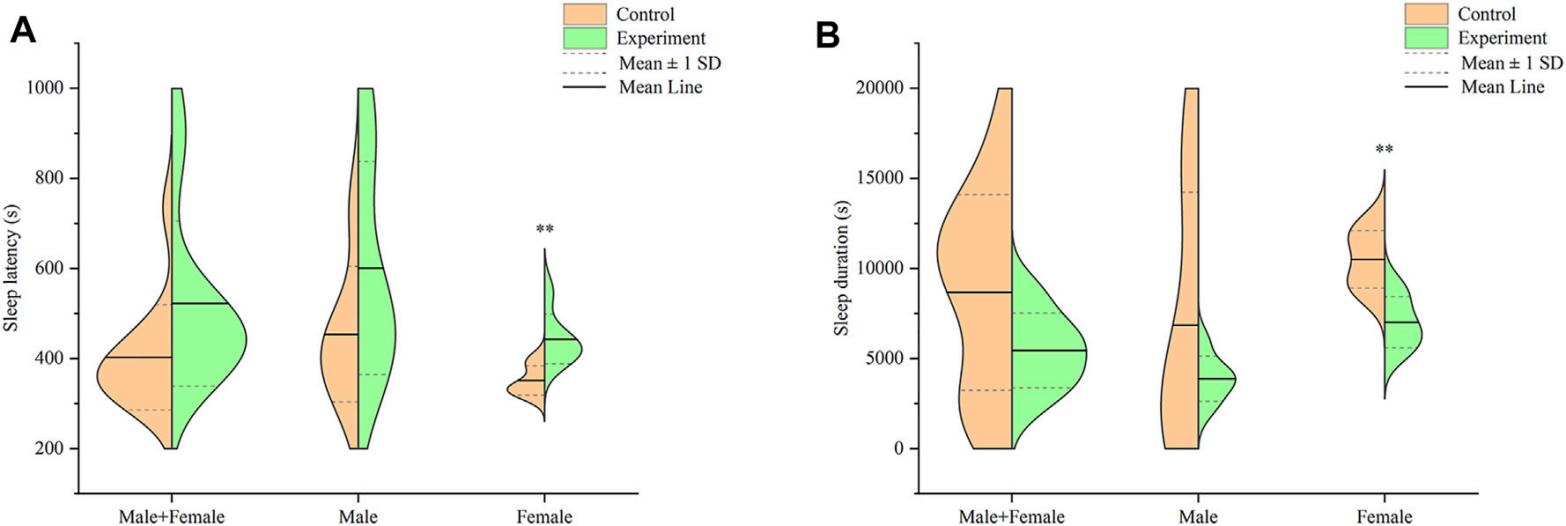
Effect of Hp-associated chronic gastritis on suprathreshold dose of sodium pentobarbital-induced sleep in mice. **(A)** Split violin plot showing the effect of Hp-associated chronic gastritis on sleep latency; **(B)** Split violin plot showing the effect of Hp-associated chronic gastritis on sleep duration. **p < .01 versus control.

Profiles of sleep latency and sleep duration in mice across both groups, after intraperitoneally injection with 55 mg/kg sodium pentobarbital, are shown in Figure 6. Analysis of sleep latency revealed longer latency in mice in the model group, relative to the control, although these were not significantly different (*p* > .05) (Figure 6A). Particularly, male mice in the model group exhibited longer sleep latency than those in the control group, albeit at no statistical significance (*p* > .05). On the other hand, female mice in the model group recorded significantly longer sleep latency relative to those in the control group (*p* < .01).

Results from a comparison of sleep durations between mice in the control and model groups are shown in Figure 6B. Notably, mice in the model group had shorter, but statistically insignificant, sleep duration than those in the control group (*p* > .05). Particularly, male mice in the model group had shorter sleep durations than their counterparts in the control group, although there was no significant difference (*p* > .05). However, female mice in the model group exhibited significantly shorter sleep duration than those in the control group (*p* < .01).

## Discussion

Hp-associated chronic gastritis has become a common clinical disease (Leja et al., 2019), due to effect from both objective environment and human factors (Venneman et al., 2018; Kotilea et al., 2019). The disease causes great pain and seriously affects quality of life in people, thus requires further explorations.

Numerous reports have described establishment of Hp associated-chronic gastritis animal models (Miszczyk et al., 2014; Gonciarz et al., 2020). In this study, we adopted C57BL/6 mice which are characterized by relatively easy colonization of Hp for model establishment (Dey et al., 2021), then appropriately neutralized gastric acid with NaHCO_3_ to adjust to a weak acid environment suitable for Hp colonization (Miederer et al., 1996). Finally, we administered Hp SS1 strain with strong colonization and virulence into the stomach and successfully established a Hp-associated model (Lee et al., 1997). Previous studies using the urease test have shown that the intensity and speed of redness depend on the amount of Hp colonization, and Hp concentration must be greater than 10^5^ cfu/ml for the red color to develop (Seo et al., 2015; Uotani et al., 2015). Therefore, failure of tissues to show red does not indicate absence of Hp infection, but may imply that the concentration of Hp colonization is lower than 10^5^ cfu/ml, which is below the detection limit of the urease test. In the present study, 70.8% of mice in the model group exhibited positive results after the urease test, indicating that the Hp colonization concentration was greater than 10^5^ cfu/ml. In addition, HE staining revealed chronic inflammatory changes in the gastric mucosa of mice in the model group. The profile of Hp colonization in gastric tissue, coupled with inflammatory pathological changes, indicated that the model was successfully established.

In contrast to human beings, mice sleep during the day and act at night (Boggs et al., 2017; Filon et al., 2020). Previous studies have demonstrated the importance of an autonomous activity tester in monitoring autonomous activity of animals (Geresu et al., 2016). In this study, the purpose of autonomous activity detection was to observe the effects of Hp associated gastritis on general behavioral activities of mice during the day. The results revealed differences in counts of autonomous activity between males and females. Particularly, male mice in the model group exhibited higher (but statistically insignificant) autonomous activity than those in the control, although this trend decreased for females in the model group (*p* > .05). In addition, both male and female mice in the model group recorded lower forelimb lift counts than those in the control group. Notably, we found statistically significant differences in females, but not in males. Although forelimb lift has been used as an exploratory rearing behavior, its application as measure of anxiety is controversial. For example, some studies have shown that exploratory rearing behaviors are positively correlated with anxiety (Ennaceur, 2014), while others have reported a inexact correlation (Costall et al., 1989). Therefore, it is difficult to distinguish exactly whether Hp-associated chronic gastritis mice have the same anxiety phenotype as HP-associated chronic gastritis patients (Buzás 2006; Takeoka et al., 2017; Kim et al., 2020).

Sodium pentobarbital is a Gamma Absorptiometry Aminobutyric Acid (GABAA) receptor agonist. Notably, activation of GABAA receptor has been associated with sedative, hypnotic and anticonvulsant effects (Choi et al., 2014). Analysis of sodium pentobarbital-induced sleep is one of the most commonly used methods for detection of sleep quality in animals (Um et al., 2021). In this study, we employed this method for analysis of sleep quality in mice, and found that Hp-associated chronic gastritis caused poor sleep quality in mice, especially females. Particularly, male mice in the model group exhibited longer and shorter (but statistically insignificant) sleep latency and sleep duration, respectively, than those in the control group. On the other hand, female mice in the model group recorded significantly longer sleep latency and shorter sleep duration than their counterparts in the control group. These results are consistent with findings from previous clinical reports that found poor sleep quality in patients with Hp-associated chronic gastritis (Buzás 2006). However, specific features of sleep disturbance and gender-based differences in Hp-infected patients have not been reported necessitating further research. It has been reported that Hp is positively correlated with obstructive apnea syndrome (Unal et al., 2003), and the sleep quality of Hp-infected patients could be improved with the eradication of Hp and the relief of chronic gastritis symptoms (Olafsson et al., 2002).

Previous studies have shown that autonomous activity and sleep-wake rhythm are regulated by the nervous system (Mišić et al., 2016; Oikonomou et al., 2019; Jackson et al., 2020; Krone et al., 2021) and Hp infection might directly or indirectly affect the nervous system via the microbiome-gut-brain axis (Gorlé et al., 2021). Vacuolating cytotoxin A (VacA) is a major cytotoxin produced by Hp in the stomach (Cover et al., 2005). VacA might travels *via* the peripheral circulation, passes through the blood-brain barrier (BBB) and affects entire brain including hypothalamus (Suzuki et al., 2019). Helicobacter pylori infection can lead to chronic inflammation (Abadi et al., 2017), and various inflammatory mediators, produced locally in the stomach, such as interleukin (IL) −1β, 6, −8, −10, and −12, tumor necrosis factor (TNF) and interferon (IFN)γ, might reach the circulation and induce neuroinflammation and toxicity (Peek et al., 2010; Alvarez-Arellano et al., 2014). The effects of Hp infection, discussed above, may lead to neurological disorders (Budzyński et al., 2014; Gorlé et al., 2021), and that may be the cause of abnormal activity and sleep in mice. To date, however, the specific mechanism remains unclear, due to lack of research on the subjects. This study lays a foundation for future relevant experimental research. Therefore, further explorations are required to elucidate the specific mechanism underlying effect of Hp-associated chronic gastritis on autonomous activity and sleep quality.

## Conclusion

Results of the present study indicated that Hp-associated chronic gastritis affects autonomous activity and sleep quality in mice in a gender-dependent manner. Specifically, male mice with Hp-associated chronic gastritis exhibit higher activity, lower forelimb lift counts, prolonged sleep latency and shorter sleep duration relative to healthy controls, albeit with no statistical significance. Conversely, female mice with Hp-associated chronic gastritis showed lower activity (albeit at no statistical significance), but significantly lower forelimb lift counts, prolonged sleep latency, and shortened sleep duration, relative to those in the control group. Overall, our results indicate that Hp-associated chronic gastritis affects autonomous activity and sleep quality of mice, consistent with previous reports that have associated the condition with poor sleep quality in patients. However, the specific mechanism underlying this effect remains unknown, thus further explorations are needed. Based on our results, we recommend use of female mice as model animals for future experimental studies.

## Data Availability

The original contributions presented in the study are included in the article/Supplementary Material, further inquiries can be directed to the corresponding author.
